# High Gain and Broadband Absorption Graphene Photodetector Decorated with Bi_2_Te_3_ Nanowires

**DOI:** 10.3390/nano11030755

**Published:** 2021-03-17

**Authors:** Tae Jin Yoo, Wan Sik Kim, Kyoung Eun Chang, Cihyun Kim, Min Gyu Kwon, Ji Young Jo, Byoung Hun Lee

**Affiliations:** 1 Center for Semiconductor Technology Convergence, Department of Electrical Engineering, Pohang University of Science and Technology, 77 Cheongam-ro, Nam-gu, Pohang, Gyeongbuk 37673, Korea; tjyoo123@postech.ac.kr (T.J.Y.); cihyun@postech.ac.kr (C.K.); 2 School of Materials Science and Engineering, Gwangju Institute of Science and Technology, 123 Cheomdangwagi-ro, Buk-gu, Gwangju 61005, Korea; kimws@gm.gist.ac.kr (W.S.K.); seoritae@icloud.com (K.E.C.); kyu817817@gist.ac.kr (M.G.K.); jyjo@gist.ac.kr (J.Y.J.)

**Keywords:** chemical vapor deposition (CVD) graphene, photodetector, Bi_2_Te_3_ nanowires, infrared photodetector, graphene photodetector

## Abstract

A graphene photodetector decorated with Bi_2_Te_3_ nanowires (NWs) with a high gain of up to 3 × 10^4^ and wide bandwidth window (400–2200 nm) has been demonstrated. The photoconductive gain was improved by two orders of magnitude compared to the gain of a photodetector using a graphene/Bi_2_Te_3_ nanoplate junction. Additionally, the position of photocurrent generation was investigated at the graphene/Bi_2_Te_3_ NWs junction. Eventually, with low bandgap Bi_2_Te_3_ NWs and a graphene junction, the photoresponsivity improved by 200% at 2200 nm (~0.09 mA/W).

## 1. Introduction

Graphene has attracted much interest for optoelectronic applications due to its unique photonic properties, such as its broad bandwidth absorption, short carrier lifetime, and high gain by the carrier multiplication process [1,2,3,4,5,6]. The unique photonic properties of graphene mean that graphene is utilized in various optoelectronic devices, infrared sensors, optical interconnects [7], and motion detectors. However, the low photoresponsivity due to the limited light absorption rate for a single graphene sheet (πα = 2.3%) is a drawback limiting the practical applications [8,9,10].

Therefore, alternative approaches combining a graphene photodetector with additional light absorption materials have been reported [11,12,13,14]. Various absorber materials have been investigated, including quantum dots (QDs), plasmonic metal nanoparticles, perovskites, and organic complexes [11,12,13,14]. Small bandgap semiconductor material such as PbS (0.83 eV) is useful for extending the bandwidth to the near-infrared region [15]. In addition, colloidal QDs are used to obtain a high photo gain at specific wavelengths. For example, PbS QDs decorating a graphene photodetector yielded a 10^7^ A/W photoresponsivity at 895 nm [12]. Similarly, when metal nanoparticles were utilized on the graphene channel to improve the photoabsorption using the plasmonic effect, a 6.1 mA/W of photoresponsivity was obtained at 514 nm [11]. Recently, a few studies have been carried out with perovskites and organic complexes as an absorber to improve the absorption in visible wavelengths. In the case of the perovskite-graphene photodetector, the photoresponsivity was 180 A/W at 520 nm [13]. In the organic-graphene photodetector, a photoresponsivity of ~10^6^ A/W was obtained in the 600–1500 nm region [14].

However, when an absorber is placed on a graphene channel, it limits the applicable wavelength because the bandgap of an absorber determines the photon absorption range. In addition, in an organic-based absorber layer, the time constant is approximately hundreds of microseconds due to the low conductivity and charge trapping. Moreover, when a high-temperature process over 1000 ℃ or chemical coating process is used, carbon decomposition or residual chemical doping can affect the electronic properties of graphene. Therefore, the absorber and deposition process should be chosen very carefully. 

In this sense, low bandgap Bi_2_Te_3_ may represent a good absorber candidate for a graphene photodetector. The bandgap of Bi_2_Te_3_ is 0.15–0.3 eV [16], which can absorb up to mid-infrared wavelengths and be fabricated in the form of nanowires [17,18], nanoplates [19], and nanosheets [18]. Gao et al. reported a graphene photodetector decorated with Bi_2_Te_3_ nanoplates, which was grown by thermal chemical vapor deposition (CVD) and achieved a 35 A/W photoresponsivity with a gain of up to 83 at 532 nm [19]. However, the limited coverage on the graphene channel and high-temperature CVD process can induce cracks or wrinkles due to the difference in the thermal expansion coefficient between two materials [20]. Moreover, the electronic properties of graphene are degraded during the high-temperature CVD process for Bi_2_Te_3_. Furthermore, the photoconductive gain was limited due to the low surface to volume/light absorption ratio of single-crystal Bi_2_Te_3_. 

In this work, we investigated a high photoconductive gain graphene photodetector using Bi_2_Te_3_ nanowire. The photoresponsivity was increased by 200% at 980 nm after the addition of the Bi_2_Te_3_ nanowire. The damage to the graphene was minimized using a room temperature drop-casting process, and a full area coverage on the channel was achieved. Moreover, a broadband absorption range from 400 to 2200 nm and high photoconductive gain of 3 × 10^4^ were obtained.

## 2. Materials and Methods

### Device Fabrication

**Figure 1:**
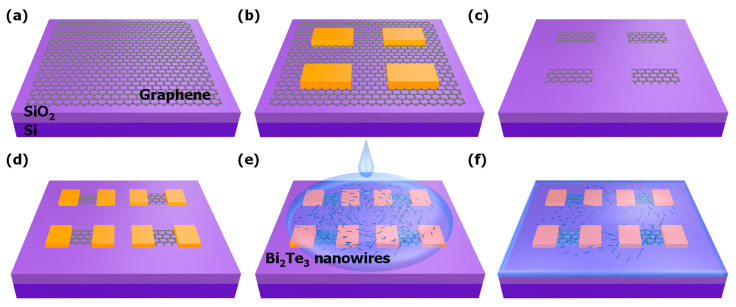
Schematic diagram of the Bi_2_Te_3_ nanowires (NWs)/graphene photodetector fabrication process. (**a**) Graphene transfer on the SiO_2_/Si substrate, (**b**) graphene channel hard mask, (**c**) graphene channel patterning, (**d**) source and drain (S/D) electrode patterning, (**e**) Bi_2_Te_3_ nanowires (NWs) drop casting on the device, and (**f**) annealing the fabricated device.

A monolayer graphene sheet (1 cm × 1 cm), grown on copper (Cu) foil by using a thermal CVD process, was transferred on the SiO_2_ (90 nm)/Si substrate using the poly(methyl methacrylate) (PMMA)-based wet transfer method (Figure 1a). The i-line (365 nm) lithography process was used to pattern the graphene channel and electrodes. The graphene channel was patterned using a positive photoresist and Au hard mask (20 nm) to prevent organic contamination from the photoresist (Figure 1b). Graphene channel patterning was carried out by O_2_ plasma Asher (100 W, 90 s), and the remaining photoresist was removed using acetone (Figure 1c). The sample was rinsed with methanol and de-ionized water for 5 min each. Then, Au metal (50 nm) was deposited using an e-beam evaporator under 10^−7^ Torr, and the source and drain electrode shape was patterned using lithography and metal wet etching, respectively (Figure 1d). To protect the graphene surface from the air and water degradation during the photodetector operation, 30 nm Al_2_O_3_ was deposited at 130 °C using the atomic layer deposition (ALD) process (300 cycles of trimethylammonium (TMA) and water source). Finally, the samples were annealed at 300 °C for 1 h in a vacuum chamber (10^−7^ Torr) [9]. On the other hand, the Bi_2_Te_3_ NWs/graphene photodetector was not passivated by Al_2_O_3_. 

Bi_2_Te_3_ NWs were prepared with the conventional polyol process [21,22] (Figure S1). The size distribution of Bi_2_Te_3_ NWs was uniformly distributed, with a standard deviation of length and diameter size of 36.01 and 6.58 nm, respectively. The average length and diameter of fabricated Bi_2_Te_3_ NWs were 611.2 and 67.3 nm, respectively. The Bi_2_Te_3_ NWs were dispersed in the ethanol solution using sonication with a value of 40 kHz for 5 min, drop cast on the graphene photodetector, and dried in a desiccator for 5 min (Figure 1e,f). 

## 3. Results and Discussion

**Figure 2:**
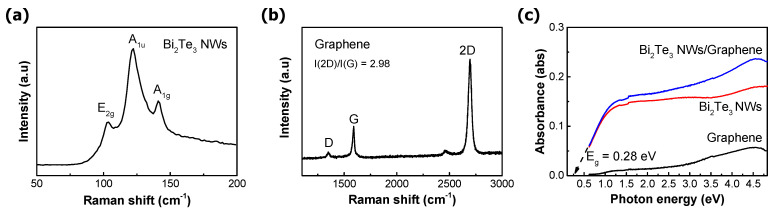
Raman spectroscopic measurements of (**a**) Bi_2_Te_3_ NWs and (**b**) graphene, and (**c**) UV-Vis absorption spectroscopy of each stack.

Figure 2a,b shows the Raman spectra of Bi_2_Te_3_ NWs and graphene channel, respectively. Three distinct Raman peaks can be observed at 102, 122, and 140 cm^−1^ (Figure 2a). These peaks indicate the E_2g_, A_1u_, and A_1g_ vibrational mode of Bi_2_Te_3_ NWs, denoting the quality of the single-crystallinity of Bi_2_Te_3_ NWs [23]. The Raman spectra for transferred graphene show three peaks at 1350, 1589, and 2695 cm^−1^ [24]; those peaks indicate the D-peak, G-peak, and 2D peak, respectively, as denoted in Figure 2b. The I(2D)/I(G) ratio of the transferred graphene layer was 2.98, which indicates single-layer graphene. Figure 2c shows the light absorbance of material stacks used in this work; graphene, Bi_2_Te_3_ NWs, and graphene/Bi_2_Te_3_ NWs in the spectral range from 0.62 to 4.6 eV. The absorbance of graphene was quite poor compared to that of Bi_2_Te_3_ NWs. The absorbance of graphene was distributed in the range of 0.057 (4.58 eV)~0.0026 (0.62 eV). The absorbance of Bi_2_Te_3_ NWs was nearly four times higher at the maximum absorption point compared to graphene. When Bi_2_Te_3_ NWs were combined with graphene, the absorbance was increased by 7.75~20.2 times in the 1.0–3.0 eV range and by ~5 times at 4.5 eV compared to the absorbance of the graphene layer alone. Even though the absorbance curve shown in Figure 2c is valid down to 0.62 eV, by extrapolating the curve near the low photon energy region (0.62–1.0 eV), the bandgap of Bi_2_Te_3_ NWs was estimated to be 0.28 eV. This value matches well with the previous result on Bi_2_Te_3_ thin film [16].

**Figure 3:**
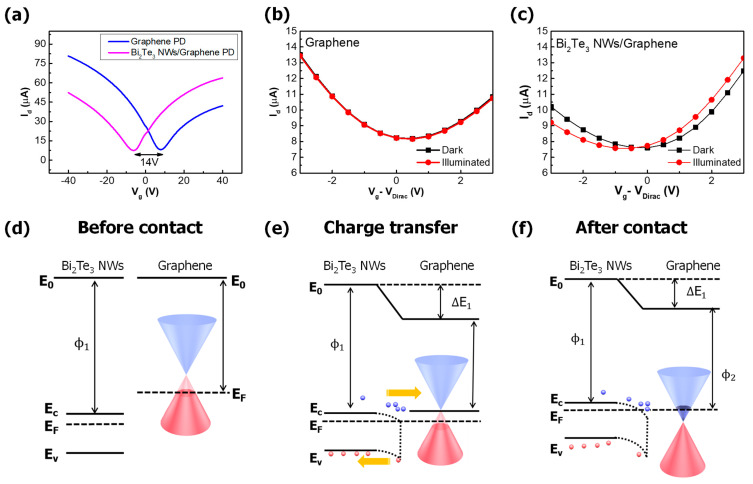
Band diagrams for graphene/Bi_2_Te_3_ NWs junction contact (**a**) before contact formation, (**b**) charge transfer during contact, and (**c**) after contact formation with Bi_2_Te_3_ NWs and graphene. E_0_ is the vacuum level; E_F_ is the Fermi level; E_c_ and E_v_ are conduction and valence bands of graphene and Bi_2_Te_3_ NWs, respectively; and ϕ_1_ and ϕ_2_ are work functions of Bi_2_Te_3_ NWs and graphene, respectively. (**d**) I_d_-V_g_ characterization of graphene only device (in blue) and Bi_2_Te_3_ NWs decorated device (in purple). (**e**,**f**) I_d_-V_g_ characterization under 980 nm illumination with an intensity of 1 mW/cm^2^.

After NW decoration on the channel, the transfer curve of the graphene photodetector exhibited a significant change due to the strong electron doping effect, indicating a −14 V Dirac voltage shift, as shown in Figure 3a. The field-effect mobility of the graphene photodetector was 1624 and 1550 cm^2^/Vs for the electron and hole, respectively, while that of the Bi_2_Te_3_ NWs/graphene device was 1058 cm^2^/Vs for the electron and 1227 cm^2^/Vs for the hole. It has been reported that the mobility can be degraded by the phonon scattering of charge carriers when graphene is in contact with substrates or stacked by other materials and by an increase of the charge trap density [25,26]. Therefore, it is speculated that the reason for the mobility degradation of the Bi_2_Te_3_ NWs/graphene device is related to the doping and surface phonon scattering due to Bi_2_Te_3_ NWs.

**Figure 4:**
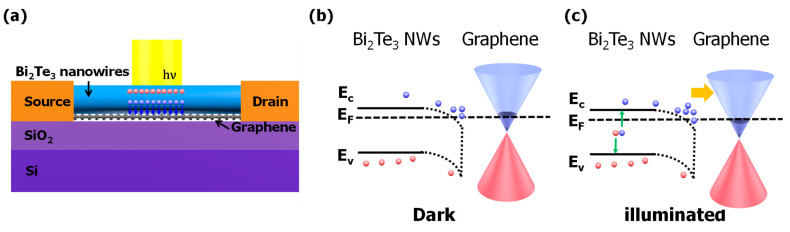
(**a**) Schematic diagram of the generation of an electron (filled circle) and a hole (open circle) under the illuminated condition for the Bi_2_Te_3_ NWs/graphene photodetector, (**b**) dark, and (**c**) illuminated status at the Bi_2_Te_3_ NWs/graphene junction.

The Dirac voltage of the graphene photodetector was nearly unaffected by 980 nm illumination, but the Dirac voltage of the Bi_2_Te_3_ NWs/graphene photodetector was shifted by −1 V, as shown in Figure 3b,c. Even though the shift under the light illumination is not significant, this shift indicates the presence of a photo-gating effect, affecting the Bi_2_Te_3_/graphene photodetector. Figure 4a shows a schematic diagram explaining the photo gating effect in the Bi_2_Te_3_/graphene photodetector [27]. When photons are incident in the Bi_2_Te_3_ NWs/graphene photodetector, the electron–hole pairs are generated in both Bi_2_Te_3_ NWs and the graphene channel and drift through the graphene channel. Since electrons and holes in the Bi_2_Te_3_ NWs have different diffusion and drift time constants, holes stay in the Bi_2_Te_3_ NWs for a longer period of time than electrons, inducing the Dirac voltage shift after illumination, as shown in Figure 4b,c. If the electron–hole pairs were generated without Bi_2_Te_3_ NWs, the recombination would occur in a short time. Therefore, the Fermi level of graphene is not significantly affected.

**Figure 5:**
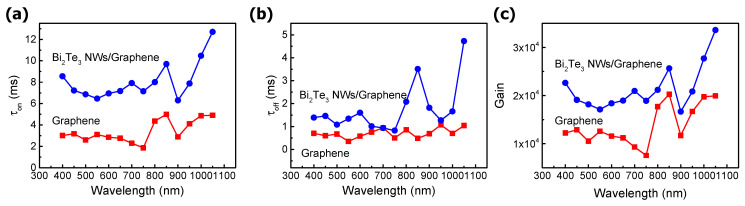
Carrier lifetime result for both the Bi_2_Te_3_ NWs/graphene and graphene only device. (**a**) τ_on_ and (**b**) τ_off_ constant. (**c**) A photoconductive gain of both device structures. The measured wavelength range is from 400 to 900 nm.

A transient photocurrent measurement with/without Bi_2_Te_3_ NW was performed to investigate the charge transfer mechanism. A Xe lamp was used as a white light source and various wavelengths from 400 to 1100 nm with a 50 nm period were applied to the device using a monochromator. The light on-off action was controlled by a chopper with a 10 Hz switching speed. During the measurement, the devices were connected to the source meter for applying bias (V_d_ = 0.01 V). The rising and falling time were calculated with the equations 1 − exp[−(t − t_on_)/τ_on_] and exp[−(t − t_off_)/τ_off_], respectively, as shown in Figure 5a,b. The transient on-off current for the extraction rising and falling time is shown in Figure S2. The currents of 10% and 90% were valid for extracting the rising (falling) time. When the photons are incident to Bi_2_Te_3_ NW decorated devices, the photocurrent generation time is increased to 8 ms, which represents a two-fold increase compared to the control group without Bi_2_Te_3_ NWs. In the case of the falling time, Bi_2_Te_3_ NW decorated devices show a longer time of 1.2 ms, compared to 0.8 ms for the control group.

In addition, the photoconductive gain of each device was calculated using the result of the rising and falling time. The following equation can be employed to calculate the photoconductive gain: G = τ/t_tr_, where τ is the lifetime; t_tr_ is the transit time, which is equal to L^2^/μV_d_; L is the channel length of the device; μ is the field-effect mobility; and V_d_ is the voltage applied to the drain. The photoconductive gain of the Bi_2_Te_3_ NWs/graphene photodetector was 3 × 10^4^ at 1100 nm, which is two-fold larger than that of the only graphene sample.

The devices with Bi_2_Te_3_ NWs decoration show a longer rising/falling time compared to the control group due to the charge exchange between Bi_2_Te_3_ NW and graphene during the light illumination. In the graphene photodetector, the photocarriers are generated in the whole graphene channel, but the photocarriers generated near the graphene/metal junction can only be collected due to the short recombination time [28]. 

**Figure 6:**
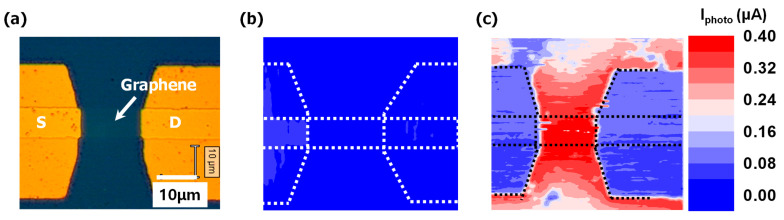
Scanning photocurrent mapping (SPCM) image of the Bi_2_Te_3_ NWs/graphene junction photodetector and graphene photodetector (**a**) optical image of the graphene photodetector device, and SPCM image of (**b**) the graphene photodetector and (**c**) Bi_2_Te_3_ NWs/graphene junction photodetector. The boundary of the source/drain metals and graphene channel are illustrated by a dotted line.

Scanning photocurrent mapping (SPCM) measurement was carried out with a 530 nm single-mode fiber laser to clarify the exact location of the photocarrier generation in our devices and compare the photocurrent generation mechanism of both the graphene photodetector and Bi_2_Te_3_ NWs/graphene photodetector. For the graphene channel device, the photocurrent was generated in the graphene/metal junction area [28]. In the case of the Bi_2_Te_3_ NWs/graphene photodetector, the photocurrent was primarily generated through the whole channel, as shown in Figure 6b,c. When Bi_2_Te_3_ NWs decorated the graphene channel, the recombination time seemed to be increased, and the whole channel could be attributed to photocurrent generation. As a result, the absorption was improved for the Bi_2_Te_3_ NWs decorated graphene photodetector. This observation confirms that the Bi_2_Te_3_ NWs improved the photocurrent generation across the graphene channel. The maximum photocurrent increased five times using Bi_2_Te_3_ NWs, from 80 to 400 nA.

**Figure 7:**
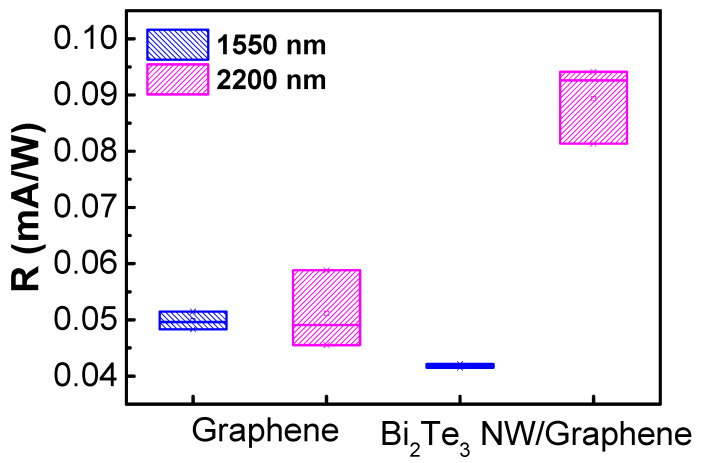
Short-wavelength infrared (SWIR) photoresponsivity comparison for both the graphene and Bi_2_Te_3_ NWs/graphene photodetector. Light power was 8 mW for a 1550 nm wavelength and 13 mW for 2200 nm. The device channel width and length were 5 and 3 μm, respectively.

Next, we examined the absorption rate and spectrum of Bi_2_Te_3_ NWs by measuring the photoresponse under an infrared illumination condition (Figure S2). The transient photoresponse measurement with near-infrared (1550 and 2200 nm) was conducted to investigate the device operation near the bandgap edge of Bi_2_Te_3_ NWs (Figure 7). The photoresponsivity of the graphene photodetector was 0.05 and 0.04 mA/W for 1550 and 2200 nm, respectively. The Bi_2_Te_3_ NWs/graphene photodetector showed a photoresponsivity similar to the graphene photodetector at 1550 nm. On the other hand, the photoresponsivity increased to 0.09 mA/W at 2200 nm as the wavelength of light became comparable to the bandgap of the Bi_2_Te_3_ NWs.

Overall, the performance of the Bi_2_Te_3_ NWs/graphene junction was improved in a wide spectral range, from visible to near-infrared. In particular, the absorption spectrum was extended to a ~2200 nm range and the photoconductive gain was improved up to 1.9 × 10^4^. The photoconductive gain of this work is comparable to that of PbS quantum dots (10^3^) [29] and drastic enhancements were achieved compared to the Bi_2_Te_3_ nanoplates/graphene junction (around 83).

## 4. Conclusions

In this work, we investigated the Bi_2_Te_3_ NWs/graphene junction using the drop-casting method. Graphene was n-doped after making contact with Bi_2_Te_3_ NWs, and the photoconductive gain was enhanced further, to 3 × 10^4^, being 1.61 times larger than that of the only graphene photodetector. Additionally, the photoresponsivity under near-infrared was improved by 200% compared to the graphene devices. Moreover, the large area solution was processible and broadband absorption was possible using this structure.

## Supplementary Materials

The following are available online at https://www.mdpi.com/2079-4991/11/3/755/s1, Figure S1. SEM image of Bi_2_Te_3_ nanowires on a substrate; Figure S2. Carrier lifetime extraction for each wavelength. The carrier lifetime of the graphene photodetector was extracted with the result of transient photocurrent measurement, the on-off frequency was 10 Hz. Figure S3. Time-photocurrent characterization under 2200 nm illumination.
