# Supplementary material for: High Gain and Broadband Absorption Graphene Photodetector Decorated with Bi2Te3 Nanowires
Source: Nanomaterials (Basel). 2021 Mar 17;11(3):755. doi: 10.3390/nano11030755 (PMC8002706; doi:10.3390/nano11030755)
Supplement: Supplementary file 1 [file nanomaterials-11-00755-s001.pdf]

Supplementary Material

High Gain and Broadband Absorption Graphene Photodetector Decorated with Bi_2Te_3 Nanowires

Tae Jin Yoo [1], Wan Sik Kim [2], Kyoung Eun Chang [2], Cihyun Kim [1], Min Gyu Kwon [2] and Ji Young Jo [2], Byoung Hun Lee [1,*]

[1] Department of Electrical Engineering, Pohang University of Science and Technology, 77, Cheongam-ro, Nam-gu, Pohang, Gyeongbuk 37673, Korea; tjyoo123@postech.ac.kr (T.J.Y.); cihyun@postech.ac.kr (C.K.)
[2] School of Materials Science and Engineering, Gwangju Institute of Science and Technology,123 Cheomdangwagi-ro, Buk-gu, Gwangju, 61005, Korea; kimws@gm.gist.ac.kr (W.S K.); seoritae@icloud.com (K.E.C.); kyu817817@gist.ac.kr (M.G.K.); jyjo@gist.ac.kr (J.Y.J.)
* Correspondence: bhlee1@postech.ac.kr ; Tel. 82-54-279-2217; Fax. 82-54-279-2903

Table of contents

1. SEM Image of Bi_2Te_3 Nanowires (NWs) Used in this Work (Figure S1) Page 2
2. Carrier Lifetime Extraction Using Monochromatic System (Figure S2) Page 3
3. Time-Photocurrent Characterization under 2200 nm Wavelength Illumination (Figure S3) Page 4

1. SEM Image of Bi_2Te_3 Nanowires (NWs) Used in this Work

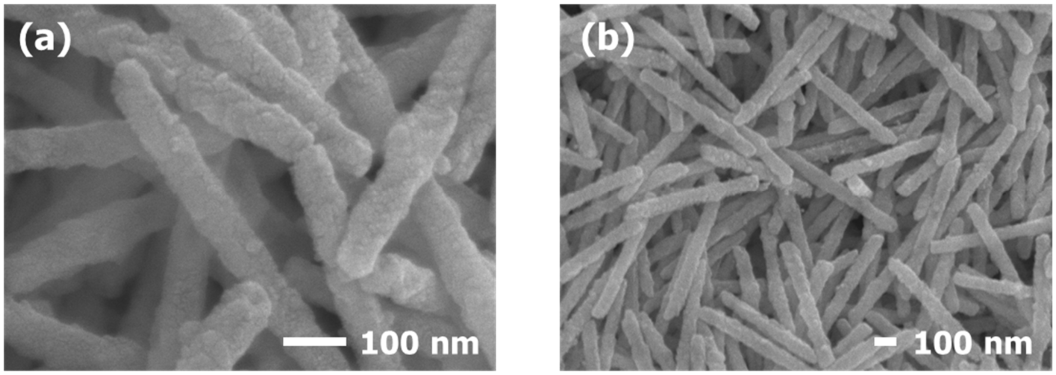

Figure 1. SEM image of Bi_2Te_3 nanowires on a substrate. (**a**) × 150,000 and (**b**) × 50,000 magnification SEM image of Bi_2Te_3 nanowires .

The average length of Bi_2Te_3 NWs is ~611.2 nm and the diameter is ~67.3 nm. The standard deviation of length and diameter size of 36.01 nm and 6.58 nm, respectively. Those numbers were characterized based on the SEM image of Bi_2Te_3 NWs on the SiO_2/Si substrate.

2. Carrier Lifetime Extraction Using Monochromatic System

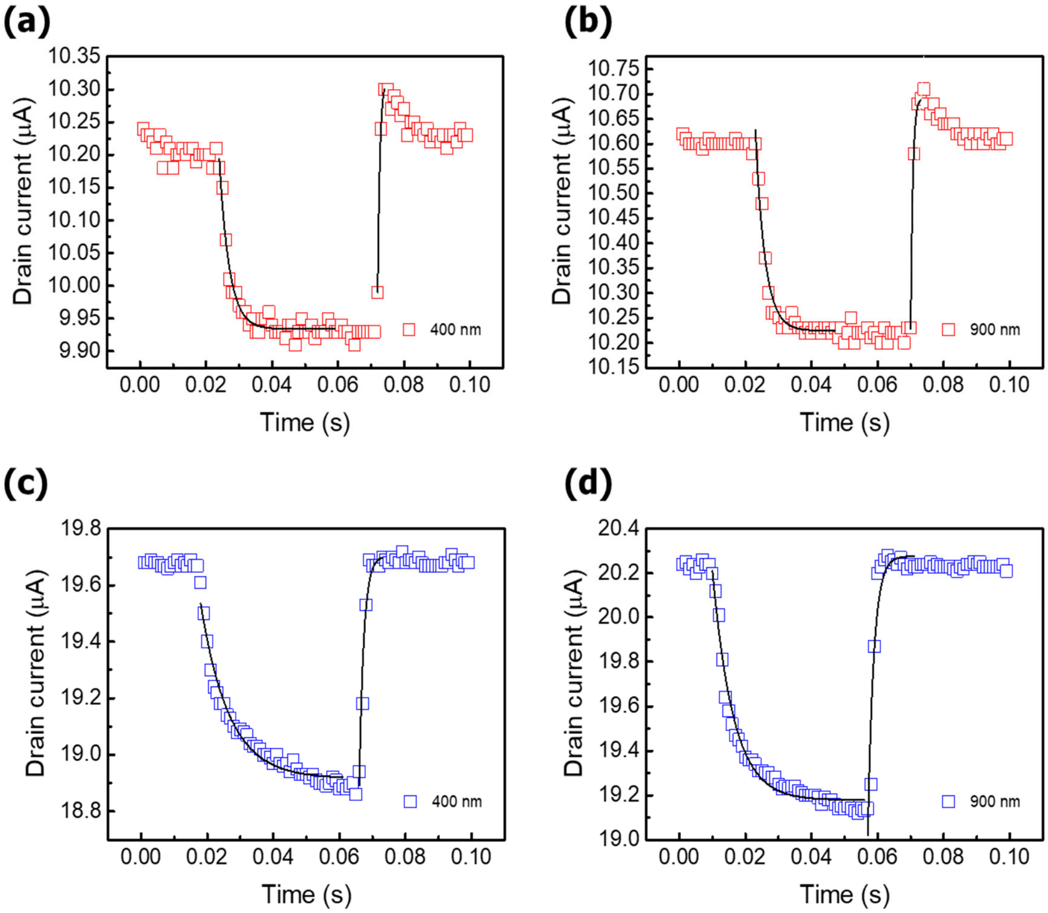

Figure S2. Carrier lifetime extraction for each wavelength**.** The carrier lifetime of the graphene photodetector was extracted with the result of transient photocurrent measurement, the on-off frequency was 10 Hz. (**a**) and (**b**) are time – photocurrent illumination result of graphene photodetector of 400 and 900 nm, res. (**c**) and (**d**) are illumination result of Bi_2Te_3 nanowires decorated graphene devices of 400 and 900 nm, respectively.

3. Time-Photocurrent Characterization under 2200 nm Wavelength Illumination

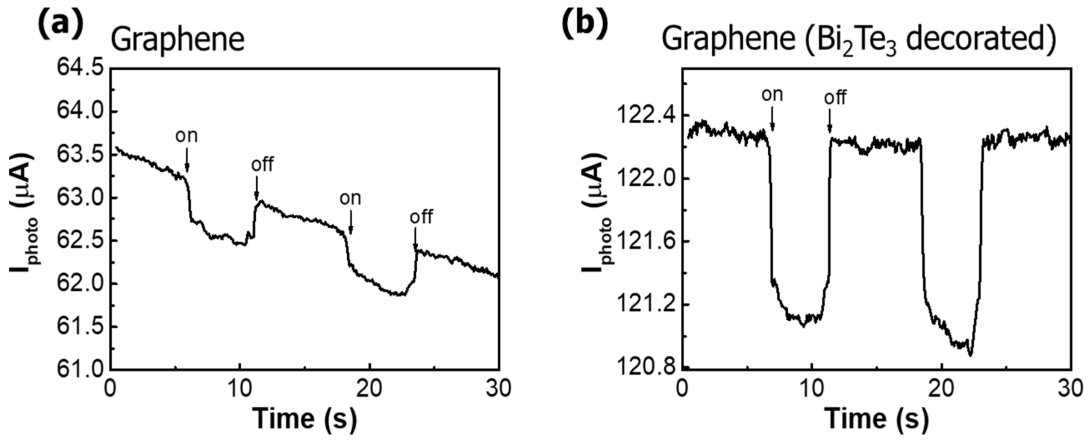

Figure 3. Time-photocurrent characterization under 2200 nm illumination. (**a**) graphene channel device, (**b**) graphene channel device decorated with Bi_2Te_3 nanowires.

Time-photocurrent characterization with 0.1 V operation condition. The period of on-off is 10 s and tried until 30 s. In graphene photodetector, there are unstable drain current degradations during the on-off cycle, and the level of drain current is two times lower compared to Bi_2Te_3 nanowire doped photodetector. In case of Bi_2Te_3 decorated graphene photodetector, it showed stable drain current level before and after on-off cycle test.
